# Salvage Liver Transplantation Is a Reasonable Option for Selected Patients Who Have Recurrent Hepatocellular Carcinoma after Liver Resection

**DOI:** 10.1371/journal.pone.0036587

**Published:** 2012-05-04

**Authors:** Zhenhua Hu, Jie Zhou, Xiaofeng Xu, Zhiwei Li, Lin Zhou, Jian Wu, Min Zhang, Shusen Zheng

**Affiliations:** 1 Department of Hepatobiliary and Pancreatic Surgery, First Affiliated Hospital, School of Medicine, Zhejiang University, Hangzhou, China; 2 Key Laboratory of Combined Multi-Organ Transplantation, Ministry of Public Health, First Affiliated Hospital, School of Medicine, Zhejiang University, Hangzhou, China; 3 Key Laboratory of Organ Transplantation, First Affiliated Hospital, School of Medicine, Zhejiang University, Hangzhou, China; Brigham & Women's Hospital, and Harvard Medical School, United States of America

## Abstract

**Background:**

Salvage liver transplantation (SLT) has been reported as being feasible for patients who develop recurrent hepatocellular carcinoma (HCC) after primary liver resection, but this finding remains controversial. We retrospectively studied the clinical characteristics of SLT recipients and conducted a comparison between SLT recipients and primary liver transplantation (PLT) recipients.

**Methodology and Principal Findings:**

A retrospective study examined data from the China Liver Transplant Registry (CLTR) for 6,975 transplants performed from January 1999 to December 2009. A total of 6,087 patients underwent PLT and 888 patients underwent SLT for recurrence. Living donor liver transplantation (LDLT) was performed in 389 patients, while 6,586 patients underwent deceased donor liver transplantation (DDLT). Kaplan-Meier curves were used to compare survival rates. The 1-year, 3-year, and 5-year overall survival of SLT recipients was similar to that of PLT recipients: 73.00%, 51.77%, and 45.84% vs. 74.49%, 55.10%, and 48.81%, respectively (P = 0.260). The 1-year, 3-year and 5-year disease-free survival of SLT recipients was inferior to that of PLT recipients: 64.79%, 45.57%, and 37.78% vs. 66.39%, 50.39%, and 43.50%, respectively (P = 0.048). Similar survival results were observed for SLT and PLT within both the LDLT and DDLT recipients. Within the SLT group, the 1-year, 3-year, and 5-year overall survival for LDLT and DDLT recipients was similar: 93.33%, 74.67%, and 74.67% vs. 80.13%, 62.10%, and 54.18% (P = 0.281), as was the disease-free survival: 84.85%, 62.85%, and 62.85% vs. 70.54%, 53.94%, and 43.57% (P = 0.462).

**Conclusions:**

Our study demonstrates that for selected patients, SLT has similar survival to that of PLT, indicating that SLT is acceptable for patients with recurrent HCC after liver resection. Given the limited organ donor pool, salvage LDLT might be considered as a possible treatment.

## Introduction

With long-term developments in the management strategy for patients with hepatocellular carcinoma (HCC), considerable experience has been gained in the treatment of HCC patients. Liver resection has been the mainstay of surgical treatment for HCC [1]. Recurrence is the most frequent cause of treatment failure after liver resection [2]. With the development of liver transplantation in recent decades, this has now been gradually accepted as the treatment of choice [2], [3], [4]. Studies have shown superior survival results after transplantation compared with resection, especially in terms of disease-free survival rates [1], [5], [6], [7], [8]. However, in some countries where the availability of liver donors is limited, HCC is still primarily treated with liver resection, or other locoregional therapies [9], [10], [11], [12].

When HCC recurs and further treatments are no longer possible, liver transplantation may be utilized in the form of salvage liver transplantation (SLT) [10], [13], [14]. However, there have long been controversies about SLT for recurrent HCC after liver resection. It is uncertain whether the outcomes of SLT are as good as primary liver transplantation (PLT) [1], [15]. Although there has been controversy about the suitability of PLT or SLT after primary liver resection for HCC patients for more than 10 years, little published data and few reports with large patient samples from multicenters are to be found. The present study focuses on the clinical patterns of SLT recipients with recurrent HCC and compares the perioperative course and survival in patients who have undergone SLT with those who have undergone PLT.

## Methods

### Objectives

The aim of this research was to study the feasibility of SLT for patients with HCC recurrence after primary liver resection.

### Participants

The details of 17,172 liver transplants performed from January 1999 to December 2009 were collected by the China Liver Transplant Registry (CLTR) from 76 liver transplantation centers all around China.

**Figure 1:**
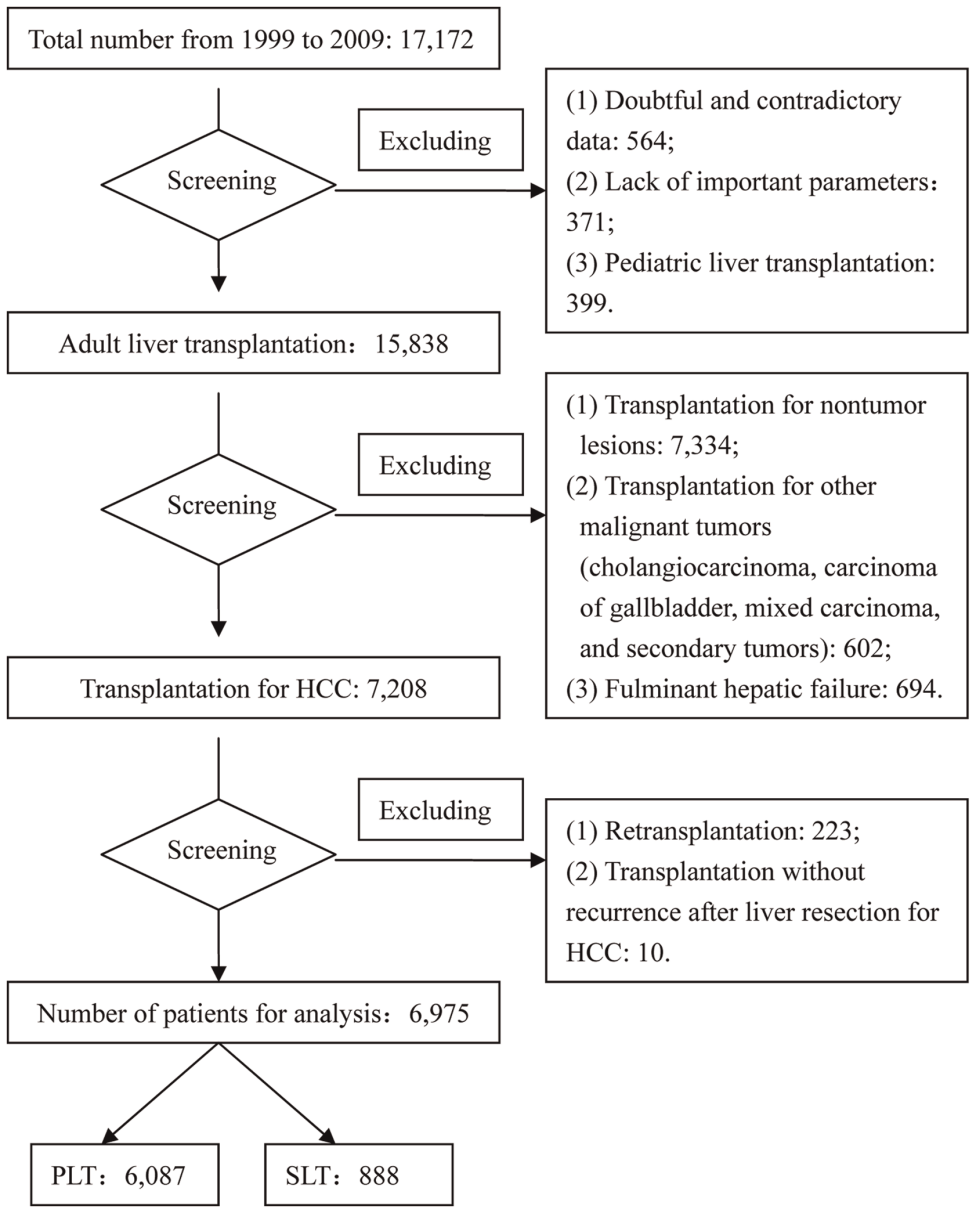
Flowchart of patients.

A total of 10,197 patients were excluded from this study using the following exclusion criteria: liver transplantation for nontumor lesions; liver transplantation for other malignant tumors (cholangiocarcinoma, carcinoma of gallbladder, mixed carcinoma, and secondary tumors); transplantation without recurrence after liver resection for HCC; pediatric liver transplantation; retransplantation. The subject selection process is depicted in Figure 1. The remaining 6,975 patients who underwent liver transplantation for HCC were analyzed.

### Procedures

The 6,975 patients were divided into two groups according to the time that they received liver transplantation: (i) patients who underwent liver transplantation when the tumor was initially discovered (PLT group; n = 6,087); (ii) patients who underwent liver transplantation for recurrent HCC after primary liver resection (SLT group; n = 888). They could also be divided into another two groups in terms of the type of graft: (i) those who underwent living donor liver transplantation (LDLT; n = 389); (ii) those who underwent deceased donor liver transplantation (DDLT; n = 6,586). Among the LDLT recipients, 360 patients (92.54%) underwent PLT and 29 patients (7.46%) underwent SLT, compared with 5,727 PLT patients (86.96%) and 859 SLT patients (13.04%) in the DDLT group (Table 1).

**Table 1:** Patient classification.

	PLT recipients	SLT recipients	Total
**LDLT recipients**	360 (180)	29 (15)	389 (195)
**DDLT recipients**	5,727 (2,651)	859 (387)	6,586 (3,038)
**Total**	6,087 (2,831)	888 (402)	6,975 (3,233)

Note: Within the parentheses is the number of recipients who met the Hangzhou criteria.

Abbreviations: PLT, primary liver transplantation; SLT, salvage liver transplantation; LDLT, living donor liver transplantation; DDLT, deceased donor liver transplantation.

A comparison was made between the PLT and SLT groups for all 6,975 patients in terms of clinical profile and overall characteristics, which included follow-up time, gender, age, underlying liver disease, type of graft, number of tumors, diameter of the largest tumor, preoperative AFP level, post-transplant macrovascular invasion, and pretransplant treatment. Operative characteristics were compared in terms of operation time, blood loss, intensive care unit (ICU) stay after transplantation, and time in hospital after transplantation. The occurrence of major postoperative complications, which included biliary, vascular, and bleeding complications, postoperative infection, intra-abdominal collection/abscess, and renal failure, were compared between the two groups.

Patients were also assessed by the Hangzhou criteria, which we have previously shown to be similar to the Milan criteria in terms of defining a good prognosis group [16]. Among the 6,975 patients, 3,233 (46.35%) met these criteria, with 2,831 (87.57%) in PLT group and 402 (12.43%) in SLT group. Among the LDLT recipients who met the criteria, 180 (92.31%) had undergone PLT compared with 15 (7.69%) who had undergone SLT. Among the DDLT recipients who met the criteria, there were 2,651 (87.26%) who had undergone PLT and 387 (12.74%) who had undergone SLT (Table 1).

In order to assess whether patients with HCC recurrence after primary liver resection had the same access to liver transplantation as patients who had not undergone a previous liver resection in terms of liver allocation, survival and disease-free survival were both calculated from the date when recipients received their liver transplant. The 1-year, 3-year, and 5-year overall and disease-free survival rates were compared first between the PLT and SLT groups for all 6,975 patients, then between PLT and SLT recipients within selected patient groups, namely those patients who met the Hangzhou criteria, the LDLT group and the DDLT group, and finally between the LDLT and DDLT recipients within the SLT group.

### Ethics statement

Ethical approval was obtained from the Committee of Ethics in Biomedical Research of Zhejiang University. Written informed consent was obtained from all participants.

The research design was hospital-based and retrospective with all cases being well evaluated. The research was approved by the CLTR.

### Statistical methods

Descriptive statistics were expressed as mean (standard deviation [SD]) or median (inter-quartile range). The Chi square test or Fisher's test, where appropriate, was used for univariate comparisons. For univariate survival analysis, plots were created and comparisons made using the Kaplan-Meier method. Differences were considered significant at P≤0.05. All statistical analyses were performed by the CLTR using SAS software, version 9.2.

## Results

### Patient profiles

Among the 6,975 HCC patients, 6,087 (87.3%) underwent PLT, while 888 (12.7%) underwent primary liver resection followed by SLT for HCC recurrence. Median follow-up in the PLT recipients was 12.40 months (inter-quartile range 3.29–28.78 months) compared with 12 .24 months in the SLT recipients (inter-quartile range 2.99–29.90 months), which was not significantly different. A total of 5,449 PLT recipients (89.52%) were male, compared with 819 (92.23%) SLT recipients (P = 0.012). There were no significant differences as regards the mean age (50.0 vs. 49.7), underlying disease (hepatitis B, 88.07% vs. 89.08%) and post-transplant macrovascular invasion (28.67% vs. 27.59%). However, significant differences were observed between PLT recipients and SLT recipients in the type of graft (5.91% LDLT and 94.09% DDLT vs. 3.27% LDLT and 96.73% DDLT, respectively); the number of tumors (median 1, range 1–2 vs. median 2, range 1–4, respectively); the diameter of the largest tumor (median 4 cm, range 2.5–7 cm vs. median 3 cm, range 2–5 cm, respectively); and the preoperative AFP level (median 134.72 ng/ml, range 13.76–1,000 ng/ml vs. median 78.23 ng/ml, range 9.13–670.75 ng/ml, respectively).

Pretransplant treatments included transcatheter arterial chemoembolization (TACE), radiofrequency ablation (RFA), systemic chemotherapy, alcohol injection, and combination treatments, all of which showed significant differences between the PLT and SLT groups (P<0.001). Patient profiles and characteristics are summarized in Table 2.

**Table 2:** Clinical profiles and overall characteristics of patients who underwent PLT and SLT for HCC.

	PLT	SLT	P-value
**Patients, number**	6,087	888	
**Follow up time** **median (inter-quartile range), months**	12.40 (3.29–28.78)	12.24 (2.99–29.90)	NS
**Gender**			
**Male, No. (%)**	5,449(89.52)	819(92.23)	0.012
**Age, mean(SD)**	50.0(9.28)	49.7(9.67)	NS
Underlying liver disease, No. (%)			NS
Hepatitis B	5,361(88.07)	791(89.08)	
Hepatitis C	426(7.00)	44(4.95)	
Idiopathic/cryptogenic cirrhosis	130(2.14)	19(2.14)	
Hepatitis, Non-A, B, C	48(0.79)	12(1.35)	
Alcoholic Liver Cirrhosis	40(0.66)	4(0.45)	
Auto-immune hepatitis	20(0.33)	4(0.45)	
Hepatitis A	6(0.10)	0(0)	
Drug induced liver injury	1(0.02)	0(0)	
Others, non-specified	55(0.90)	14(1.58)	
Graft type, No. (%)			0.001
LDLT	360(5.91)	29(3.27)	
DDLT	5,727(94.09)	859(96.73)	
**Number of tumors, median (inter-quartile range)**	1(1–2)	2(1–4)	<0.001
**Diameter of largest tumor, median (inter-quartile range), cm**	4(2.5–7)	3(2–5)	<0.001
**Preoperative AFP level, median (inter-quartile range), ng/ml**	134.72 (13.76–1,000)	78.23 (9.13–670.75)	<0.001
**Post-transplant macrovascularinvasion, No. (%)**	1,745(28.67)	245(27.59)	NS
**Pretransplant treatment, No. (%)**			<0.001
TACE	1,263(20.75)	299(33.67)	
RFA	199(3.27)	39(4.39)	
Systemic chemotherapy	60(0.99)	52(5.86)	
Alcohol injection	35(0.57)	9(1.01)	
Combination treatment	272(4.47)	148(16.67)	
None	4,258(69.95)	341(38.40)	

Note: Data are presented as number of patients (% of total patients) or median (inter-quartile range) or mean (SD).

Abbreviations: PLT, primary liver transplantation; SLT, salvage liver transplantation; HCC, hepatocellular carcinoma; LDLT, living donor liver transplantation; DDLT, deceased donor liver transplantation; TACE, transcatheter arterial chemoembolization; RFA, radiofrequency ablation; NS, not significant.

### Operative characteristics and postoperative complications

Operative characteristics of the PLT and SLT patients are summarized and compared in Table 3. The median operation time in the PLT recipients was 8 hours (inter-quartile range 6.5–9.5 hours), compared with 8 hours (inter-quartile range 7–10 hours) in the SLT recipients (P<0.001). The median blood loss during the operation in the SLT group (2,000 ml; inter-quartile range 1,200–4,000 ml) was significantly more than that in the PLT group (1,700 ml; inter-quartile range 1,000–3,000 ml; P<0.001). These differences did not translate into differences between the PLT and SLT recipients in terms of the length of ICU stay or the time in hospital after transplantation.

**Table 3:** Operative characteristics of patients undergoing PLT and SLT for HCC.

	PLT (n = 6,087)	SLT (n = 888)	P-value
**Operation time (hours)**	8(6.5–9.5)	8(7–10)	<0.001
**Blood loss (ml)**	1,700(1,000–3,000)	2,000(1,200–4,000)	<0.001
**ICU stay after transplant (hours)**	111(72–170)	108(62–182)	NS
**Time in hospital after transplant (days)**	32(24–45)	31(22–47)	NS

Note: Data are presented as median (inter-quartile range). n, number of patients.

Abbreviation: PLT, primary liver transplantation; SLT, salvage liver transplantation; HCC, hepatocellular carcinoma; ICU, intensive care unit; NS, not significant.

Postoperative complications were compared for all 6,975 recipients and are detailed in Table 4. No statistically significant differences existed between the PLT and SLT recipients in terms of postoperative biliary, vascular, and bleeding complications, postoperative infection, intra-abdominal collection/abscess, and renal failure.

**Table 4:** Postoperative complications of patients undergoing PLT and SLT for HCC.

Postoperative Complications, No. (%)	PLT (n = 6,087)	SLT (n = 888)	P-value
**Intra-abdominal collection/abscess** *	1,742(28.62)	273(30.74)	0.192
**Postoperative infection** †	1,678(27.57)	268(30.18)	0.105
**Biliary complications** §	681(11.19)	113(12.73)	0.178
**Intra-abdominal bleeding**	338(5.55)	59(6.64)	0.190
**Renal failure** ‡	206(3.38)	28(3.15)	0.721
**Vascular complications** **	202(3.32)	37(4.17)	0.194

Note: Data are presented as number of patients (% of total patients). n, number of patients.

* Intra-abdominal collection/abscess: intra-abdominal collection refers to ascites retention exceeding normal value accompanied by fever and proteinuria; intra-abdominal abscess includes subphrenic abscess, pelvic abscess, interintestinal abscess.

† Postoperative infection includes pulmonary infection, catheter-related sepsis, urinary tract infection, wound infection, opportunistic infection.

§ Biliary complications include anastomotic biliary strictures, intrahepatic biliary strictures, bile leakage.

‡ Renal failure includes chronic renal failure, acute renal failure and uremia (excluding renal failure accompanied by hypertension and neonatal uremia).

** Vascular complications include hepatic artery embolism, portal vein embolism, portal vein stenosis/ pylethrombosis, hepatic vein/ inferior vena cava stenosis/ embolism.

Abbreviation: PLT, primary liver transplantation; SLT, salvage liver transplantation; HCC, hepatocellular carcinoma.

### Survival analysis

The 1-year, 3-year, and 5-year overall survival rates and disease-free survival rates were analyzed grouped by PLT and SLT in all 6,975 recipients. Because the pretransplant status, which included the number of tumors, diameter of the largest tumor, preoperative AFP level and the pretransplant treatments, was significantly different between the PLT and SLT groups, which may have an influence on the survival analysis, we selected the patients according to the Hangzhou criteria and within the selected patients analyzed the 1-year, 3-year and 5-year overall and disease-free survival of LDLT between PLT and SLT group, DDLT between PLT and SLT group, and SLT between LDLT and DDLT recipients.

**Figure 2:**
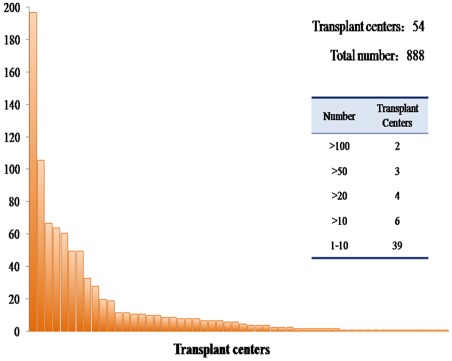
Frequency table for size of center by case volume distribution.

Because of the relatively small number of SLT patients, to eliminate any variation that might have been introduced by different centers, we also analyzed the survival rates of SLT patients by center size. A total of 54 centers which had performed SLT were included in the study; a frequency table of center size and case volume is shown in Figure 2. The 1-year, 3-year, and 5-year overall and disease-free survival rates of SLT patients from centers with SLT numbers ≤10 showed no significant differences compared with those from centers with SLT numbers >10 (79.15%, 52.91%, 41.57% vs. 71.95%, 51.52%, 45.95%, respectively, P = 0.529; and 72.30%, 45.38%, 29.47% vs. 63.47%, 45.25%, 38.16%, respectively, P = 0.262).

In the total group of 6,975 recipients, overall survival rates were similar between the PLT and SLT groups. The 1-year, 3-year, and 5-year overall survival rates were 74.49%, 55.10%, and 48.81% in PLT recipients compared with 73.00%, 51.77%, and 45.84% in SLT recipients (P = 0.260). However, a significant difference was observed in the disease-free survival rates between these two groups. The 1-year, 3-year and 5-year disease-free survival rates were 66.39%, 50.39%, and 43.50% in PLT recipients, compared with 64.79%, 45.57%, and 37.78% in SLT recipients (P = 0.048).

**Figure 3:**
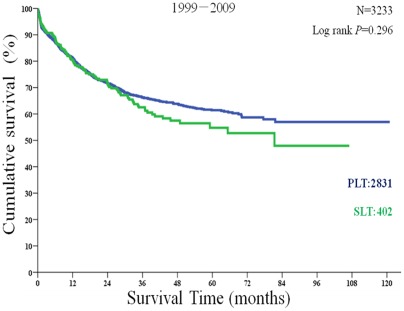
Overall survival of primary liver transplantation (PLT) and salvage liver transplantation (SLT) recipients among those patients who met the Hangzhou criteria.

**Figure 4:**
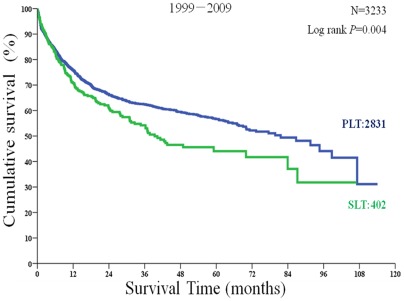
Disease-free survival of primary liver transplantation (PLT) and salvage liver transplantation (SLT) recipients among those patients who met the Hangzhou criteria.

Within selected patients of the 6,975 patients, the 1-year, 3-year, and 5-year overall survival rates were 81.11%, 66.32%, and 61.42% in PLT recipients compared with 80.59%, 62.53%, and 54.76% in SLT recipients. There was no statistically significant difference between two groups (P = 0.296; Fig. 3). However, the disease-free survival rates of PLT recipients were significantly higher than the SLT recipients at 75.83%, 62.47%, and 56.65% compared with 71.05%, 54.20%, and 44.04% (P = 0.004; Fig. 4).

Limiting the analysis to the LDLT group, both the overall and disease-free survival rates were similar between the PLT and SLT within selected patients. The 1-year, 3-year, and 5-year overall survival rates were 93.65%, 85.84%, and 85.84% in PLT recipients compared with 93.33%, 74.67%, and 74.67% in SLT recipients (P = 0.546). Disease-free survival rates were 88.92%, 78.37%, and 78.37% in PLT recipients compared with 84.85%, 62.85%, and 62.85% in SLT recipients (P = 0.214).

For the DDLT patients only, no significant difference was observed in the 1-year, 3-year, and 5-year overall survival rates between the two groups within selected patients: 80.26%, 65.14%, and 60.31% in PLT recipients and 80.13%, 62.10%, and 54.18% in SLT recipients (P = 0.417). However, the disease-free survival rates were significantly higher in the PLT group: 74.94%, 61.47%, and 55.72% in PLT recipients compared with 70.54%, 53.94%, and 43.57% in SLT recipients (P = 0.010).

Among the SLT patients only, the 1-year, 3-year, and 5-year overall survival rates within selected patients were 93.33%, 74.67%, and 74.67% in LDLT recipients compared with 80.13%, 62.10%, and 54.18% in the DDLT recipients (P = 0.281). Disease-free survival rates were 84.85%, 62.85%, and 62.85% in LDLT recipients compared with 70.54%, 53.94%, and 43.57% of in the DDLT recipients (P = 0.462). No significant differences were observed between the two groups in either the overall survival rates or disease-free survival rates.

## Discussion

Our research shows that the overall and disease-free survival of SLT recipients was similar to that of PLT recipients. For both LDLT and DDLT recipients, no survival benefit was observed for PLT. The survival rates for LDLT and DDLT in the SLT group were also similar.

With the rapid development of liver transplantation, it has now been accepted as the optimal treatment for patients with HCC because it cures both the tumor and the underlying liver disease. Mazzaferro et al. reported that the 4-year overall and recurrence-free survival after orthotopic liver transplantation was 85% and 92%, respectively, in patients who met the Milan criteria [4]. Our center has also previously reported the favorable results of recipients who met the Hangzhou criteria, with 5-year survival rates of 72.3%, which showed no significant difference from those recipients who fell within the Milan criteria [16].

However, the donor organ shortage has been a major worldwide limitation to the use of PLT for small resectable HCC [17], [18]. The long-term waiting lists due to this donor shortage may result in tumor progression, which in turn leads to potential drop-out from the waiting list [19]. In a study by Yao and colleagues, it was reported that a 6-month waiting period for liver transplantation was associated with a 7.2% cumulative probability of dropout, which increased to 37.8% and 55.1% at 12 and 18 months, respectively [20]. Del Gaudio et al. reported a better 5-year overall survival for liver resection than for liver transplantation by intention-to-treat analysis, at 66% and 58%, respectively [21]. Majno et al. assumed four main variables determining the outcome of SLT and reported the life expectancy was 8.8 years for PLT recipients compared with 7.8 years for SLT recipients, which was associated with an estimated saving of 29% of liver grafts at 5 years [15]. Under these circumstances, primary resection may be a rational way to delay tumor growth and progression while waiting for a compatible donor. SLT can be performed when HCC recurs once a donor is available.

However, there have long been controversies about SLT. In our study, among the 6,975 HCC patients who underwent liver transplantation, 6,087 patients (87.3%) underwent PLT and 888 patients (12.7%) underwent primary liver resection followed by SLT for HCC recurrence. Many more patients underwent PLT than SLT because it is known that PLT offers satisfactory survival and a high quality of life, whilst little is known about the outcomes of SLT. There are several conflicting reports of clinical experiences with SLT in the literature. The data published by Belghiti et al. indicated that both 3-year and 5-year overall survival rates in PLT and SLT recipients were similar (82% vs. 82% and 59% vs. 61%) [13]. This was confirmed by the study of Del Gaudio et al. [21] and Vennarecci et al. [22]. Conversely, Adam and colleagues reported an increased risk of recurrence (54% vs. 18%), with a poorer 5-year overall survival for SLT than for PLT (41% vs. 61%) as well as the disease-free survival (29% vs. 58%) [23].

In our analysis, the 1-year, 3-year, and 5-year overall survival rates for PLT recipients and SLT recipients showed no significant difference. With regards to disease-free survival, SLT had a higher rate of recurrence. Significant differences were observed in the median number of tumors, median diameter of the largest tumor, and median preoperative AFP level between the PLT and SLT recipients. This can be largely attributed to the strict surveillance after resection for early detection of intrahepatic recurrence in the eventual SLT recipients, which is not seen in PLT recipients. In addition, we observed a difference in pretransplant treatments between the PLT and SLT recipients, which was possibly due to two factors. Firstly, some centers used TACE and systemic chemotherapy as the post-resection procedure of choice, and when recurrence was observed, these treatments continued to be given. Secondly, RFA and alcohol injection are theoretically more likely to be effective when used in patients with early-stage tumors, which are more often observed in SLT recipients who have a lower number of tumors, smaller diameter of the largest tumor, and lower preoperative AFP level. All of these may have an influence on the survival analysis. As a result of this, the survival rates within selected patient groups were also analyzed. Our results showed that despite the difference in the pretransplant status, the overall survival of PLT recipients was similar to that of the SLT recipients, although the SLT recipients had a higher rate of recurrence. This is probably because these patients have a longer time interval of HCC from the diagnosis to liver transplantation [23].

In fact, for a proportion of patients with early HCC, especially peripheral lesions, primary liver resection can help keep the patients alive without recurrence. Even for those patients with a high risk of recurrence, close follow-up and frequent surveillance after primary liver resection can help to detect the majority of hepatic recurrences after resection at an early tumor stage, and the results of SLT are similar to those of PLT [13], [24]. However, for those patients with inadequate liver reserve, PLT may be a preferable treatment option [25].

Other issues are that a previous liver resection is likely to create adhesions and increase surgical difficulty for the potential SLT. More blood loss and longer operation time were observed in our SLT group, which demonstrated the complexity of the SLT procedure. However, there was no significant difference in either the total time in hospital or ICU stay after transplantation, which indicated that recovery from SLT is the same as from PLT. Vennarecci et al. [22] and Belghiti et al. [13] also reported that the operative time, blood loss, ICU stay, and inpatient hospital stay were similar between PLT and SLT groups. Moreover, we believe that the operation time and blood loss can be further controlled with the accumulation of experience as the number of cases of SLT increases.

The results of our study also showed that SLT did not increase the incidence of postoperative complications that are closely related to the surgical procedure, which indicates that SLT is a technically feasible procedure compared with PLT. Recent studies have revealed that laparoscopic liver resection facilitated the liver transplantation procedure compared with open liver resection in respect of reductions in operative time, blood loss, and transfusion requirements and have concluded that laparoscopic liver resection, when feasible, may be preferred to open liver resection in potential transplant candidates [19].

LDLT offers a great opportunity with regard to the supply of transplantable organs, as the prolonged waiting period and drop-out risk can be eliminated. The Markov model of Sarasin and colleagues demonstrated that patients with HCC would gain in life expectancy and cost-effectiveness from LDLT when waiting more than 7 months for a cadaveric organ [26]. Nonetheless, Hwang et al. and Lo et al. have reported contradictory results. The former observed similar survival after LDLT and DDLT (3-year survival 91.4 vs. 89.9% within the Milan criteria) [27], while the latter reported the HCC recurrence was higher after LDLT, which they believed was associated with selection bias [28]. Also, the Hwang group noted similar survival after salvage LDLT compared with primary LDLT within the Milan criteria, with 1-year, 3-year, and 5-year survival rates of 80%, 80%, and 80% compared with 87.8%, 80.1%, and 74.8%, respectively [14].

In our study, LDLT, which made up 5.91% of the total PLT recipients, made up only 3.27% of the total SLT recipients in the same period, which suggested that many surgeons are concerned about adopting LDLT for SLT. This is partially because split liver from living donors, which results in the activation of signaling pathways associated with tumor invasion, might promote growth of residual tumor cells [29], which eventually leads to recurrence. Our study has shown that for LDLT within selected patients, although those patients who received SLT also had a higher rate of recurrence, there was no statistical difference in overall survival rates and disease-free survival rates between the PLT and SLT recipients. In addition, the 1-year, 3-year, and 5-year overall and disease-free survival rates within selected patients were also similar between the LDLT and DDLT in the SLT group, which indicates that salvage LDLT is a safe procedure for selected patients. Therefore, given the critical shortage of cadaveric donor livers, LDLT is considered to be a justifiable treatment option.

Our study was a retrospective one, which shares the limitations typically associated with analyses of observational data, primarily related to the depth and quality of data available to the CLTR. Because the study was not randomly assigned, there is potential for unmeasured patient characteristics to confound the results. Although prospective, randomized, multicenter trials remain the gold standard for clinical studies, we cannot unfortunately perform such an analysis.

Another limitation is the lack of adequate information to compare those patients who received non-transplant therapies, such as secondary resection or RFA after HCC recurrence, with the SLT recipients who eventually received liver transplantation after recurrence. Therefore, we cannot assess possible differences in prognosis following these treatments. Of note, previous studies have already reported the superior survival results of PLT compared with non-transplant therapies, and in our analysis the survival of SLT recipients was similar to the PLT recipients. Therefore, we might presume that the survival of SLT patients would also be superior to non-transplant therapies. In addition, our study mainly focused on the clinical feasibility of SLT in comparison with PLT, so this may not have a significant influence on the final survival analysis.

Despite these limitations, our study represents a much larger and more comprehensive assessment of SLT for patients with HCC recurrence after primary liver resection than those previously published. Moreover, if possible we will in future assess the post-recurrence treatments based on the data from multiple centers to determine whether these have an impact on the survival of patients who undergo primary liver resection, therefore providing better guidance in the decision-making process to improve outcome for these patients.

In conclusion, this study shows that for selected patients, SLT for recurrent HCC after liver resection is a safe procedure with similar survival rates to PLT. SLT might be accepted as the treatment of choice for patients with recurrent HCC. The use of living or deceased donors does not affect survival in SLT, and living donor SLT may be a good alternative option because of the shortage of deceased donor organs.

## References

[1] Poon RT, Fan ST, Lo CM, Liu CL, Wong J (2002). Long-term survival and pattern of recurrence after resection of small hepatocellular carcinoma in patients with preserved liver function: implications for a strategy of salvage transplantation.. Ann Surg.

[2] Fan ST, Cheung ST, Lo CM (2000). Indications for liver transplantation in patients with chronic hepatitis B and C virus infection and hepatocellular carcinoma.. J Gastroenterol Hepatol.

[3] Mor E, Kaspa RT, Sheiner P, Schwartz M (1998). Treatment of hepatocellular carcinoma associated with cirrhosis in the era of liver transplantation.. Ann Intern Med.

[4] Mazzaferro V, Regalia E, Doci R, Andreola S, Pulvirenti A (1996). Liver transplantation for the treatment of small hepatocellular carcinomas in patients with cirrhosis.. N Engl J Med.

[5] Dmitrewski J, El-Gazzaz G, McMaster P (1998). Hepatocellular cancer: resection or transplantation.. J Hepatobiliary Pancreat Surg.

[6] Michel J, Suc B, Montpeyroux F, Hachemanne S, Blanc P (1997). Liver resection or transplantation for hepatocellular carcinoma? Retrospective analysis of 215 patients with cirrhosis.. J Hepatol.

[7] Bismuth H, Chiche L, Adam R, Castaing D, Diamond T (1993). Liver resection versus transplantation for hepatocellular carcinoma in cirrhotic patients.. Ann Surg.

[8] Figueras J, Jaurrieta E, Valls C, Ramos E, Serrano T (2000). Resection or transplantation for hepatocellular carcinoma in cirrhotic patients: outcomes based on indicated treatment strategy.. J Am Coll Surg.

[9] Eguchi S, Hidaka M, Tomonaga T, Miyazaki K, Inokuma T (2009). Actual therapeutic efficacy of pre-transplant treatment on hepatocellular carcinoma and its impact on survival after salvage living donor liver transplantation.. J Gastroenterol.

[10] Eguchi S, Kanematsu T, Arii S, Okazaki M, Okita K (2008). Comparison of the outcomes between an anatomical subsegmentectomy and a non-anatomical minor hepatectomy for single hepatocellular carcinomas based on a Japanese nationwide survey.. Surgery.

[11] Takayasu K, Arii S, Ikai I, Omata M, Okita K (2006). Prospective cohort study of transarterial chemoembolization for unresectable hepatocellular carcinoma in 8510 patients.. Gastroenterology.

[12] Livraghi T, Meloni F, Di Stasi M, Rolle E, Solbiati L (2008). Sustained complete response and complications rates after radiofrequency ablation of very early hepatocellular carcinoma in cirrhosis: Is resection still the treatment of choice?. Hepatology.

[13] Belghiti J, Durand F (2007). Hepatectomy vs. liver transplantation: a combination rather than an opposition.. Liver Transpl.

[14] Hwang S, Lee SG, Moon DB, Ahn CS, Kim KH (2007). Salvage living donor liver transplantation after prior liver resection for hepatocellular carcinoma.. Liver Transpl.

[15] Majno PE, Sarasin FP, Mentha G, Hadengue A (2000). Primary liver resection and salvage transplantation or primary liver transplantation in patients with single, small hepatocellular carcinoma and preserved liver function: an outcome-oriented decision analysis.. Hepatology.

[16] Zheng SS, Xu X, Wu J, Chen J, Wang WL (2008). Liver transplantation for hepatocellular carcinoma: Hangzhou experiences.. Transplantation.

[17] Bismuth H, Majno PE, Adam R (1999). Liver transplantation for hepatocellular carcinoma.. Semin Liver Dis.

[18] Strong RW (2000). Transplantation for liver and biliary cancer.. Semin Surg Oncol.

[19] Laurent A, Tayar C, Andreoletti M, Lauzet JY, Merle JC (2009). Laparoscopic liver resection facilitates salvage liver transplantation for hepatocellular carcinoma.. J Hepatobiliary Pancreat Surg.

[20] Yao FY, Bass NM, Nikolai B, Merriman R, Davern TJ (2003). A follow-up analysis of the pattern and predictors of dropout from the waiting list for liver transplantation in patients with hepatocellular carcinoma: implications for the current organ allocation policy.. Liver Transpl.

[21] Del Gaudio M, Ercolani G, Ravaioli M, Cescon M, Lauro A (2008). Liver transplantation for recurrent hepatocellular carcinoma on cirrhosis after liver resection: University of Bologna experience.. Am J Transplant.

[22] Vennarecci G, Ettorre GM, Antonini M, Santoro R, Maritti M (2007). First-line liver resection and salvage liver transplantation are increasing therapeutic strategies for patients with hepatocellular carcinoma and child a cirrhosis.. Transplant Proc.

[23] Adam R, Azoulay D, Castaing D, Eshkenazy R, Pascal G (2003). Liver resection as a bridge to transplantation for hepatocellular carcinoma on cirrhosis: a reasonable strategy?. Ann Surg.

[24] Margarit C, Escartin A, Castells L, Vargas V, Allende E (2005). Resection for hepatocellular carcinoma is a good option in Child-Turcotte-Pugh class A patients with cirrhosis who are eligible for liver transplantation.. Liver Transpl.

[25] Facciuto ME, Koneru B, Rocca JP, Wolf DC, Kim-Schluger L (2008). Surgical treatment of hepatocellular carcinoma beyond Milan criteria. Results of liver resection, salvage transplantation, and primary liver transplantation.. Ann Surg Oncol.

[26] Sarasin FP, Majno PE, Llovet JM, Bruix J, Mentha G (2001). Living donor liver transplantation for early hepatocellular carcinoma: A life-expectancy and cost-effectiveness perspective.. Hepatology.

[27] Hwang S, Lee SG, Joh JW, Suh KS, Kim DG (2005). Liver transplantation for adult patients with hepatocellular carcinoma in Korea: comparison between cadaveric donor and living donor liver transplantations.. Liver Transpl.

[28] Lo CM, Fan ST, Liu CL, Chan SC, Ng IO (2007). Living donor versus deceased donor liver transplantation for early irresectable hepatocellular carcinoma.. Br J Surg.

[29] Man K, Lo CM, Xiao JW, Ng KT, Sun BS (2008). The significance of acute phase small-for-size graft injury on tumor growth and invasiveness after liver transplantation.. Ann Surg.

